# Contact Electrification of Biological and Bio-Inspired Adhesive Materials on SiO_2_ Surfaces: Perspectives from DFT Calculations

**DOI:** 10.3390/biomimetics7040216

**Published:** 2022-11-28

**Authors:** Jing Tao, Linfeng Wang, Kaixuan Kong, Minhao Hu, Zhendong Dai

**Affiliations:** 1 Jiangsu Provincial Key Laboratory of Bionic Functional Materials, College of Mechanical and Electrical Engineering, Nanjing University of Aeronautics and Astronautics, Nanjing 210016, China; 2 College of Aerospace Engineering, Nanjing University of Aeronautics and Astronautics, Nanjing 210016, China

**Keywords:** contact electrification, polymer, first-principles calculation, bio-inspired adhesive materials

## Abstract

In this study, we investigate the contact electrification properties of glycine, cysteine, and dimethyl siloxane on silicon dioxide (SiO_2_) surfaces using density functional theory calculations. Molecule contacting through the sulfhydryl group has stronger adhesion to the SiO_2_-O and SiO_2_-OH surfaces. The SiOH/SiO_2_-Si system has the largest adhesion energy in all molecule/SiO_2_-Si contact systems and charge transfers from the molecule to the SiO_2_-O and SiO_2_-Si surfaces. The molecule/SiO_2_-OH systems have a reverse charge transfer direction. Molecules with their sulfhydryl and hydroxyl groups facing the SiO_2_-O and SiO_2_-OH surfaces have more transferred charges. The NH_2_/SiO_2_-Si system has a larger transferred charge than other molecule/SiO_2_-Si systems. The direction of charge transfer is determined by the Bader charge of the isolated surface atoms. The respective energy difference in the lowest unoccupied occupied molecular orbitals between contacting atoms influences the charge transfer. The respective energy difference in the highest occupied molecular orbitals reflects the electron attraction and affects charge transfer. Finally, the quantitative relationship between the transferred charge and energy gaps is established to evaluate the charge transfer. The findings propose a new perspective and in-depth understanding of contact electrification and shed light on the bio-inspired adhesive materials design and fabrication for engineering applications.

## 1. Introduction

Geckos have the extraordinary motion ability to temporarily and reversibly adhere to nearly all surface topographies [1,2,3]. The adhesive mechanism of geckos is believed to be dominated by the van der Waals interactions between the submicron-sized spatulae and the substrate [4,5]. By mimicking the outstanding adhesive capabilities of the gecko, researchers have fabricated different kinds of gecko-inspired artificial adhesives based on polymer materials (e.g., polyvinylsiloxane, polyurethane, polydimethylsiloxane) [6,7,8,9]. However, the mechanism of gecko adhesion and the physical origin of the adhesion-induced surface properties have been subjects of debate, especially for the improvement of the adhesion of bionic materials [10,11]. Meanwhile, it is well known that contact electrification is one kind of surface effect and almost all substances exhibit a contact electrification effect in daily life, which cannot be ignored in the study of interface adhesion. Izadi et al. measured the tribocharge and shearing forces of geckos’ footpads and found that interfacial contact electrification contributes effectively to gecko adhesion [12]. Song et al. synchronously measured interfacial tribocharge and contact forces and found that the tribocharge at the footpads of free locomotion geckos is related to contact forces [13]. In addition, geckos’ hairy attachments are easily positively tribo-charged, and thus electrostatic interaction is increasingly invoked to elucidate the adhesion of geckos [11,14]. Hence, we need to consider the presence and contribution of contact electrification in the adhesion effects of gecko and gecko-inspired materials.

Given the perspective above, what causes the contact electrification of gecko setae and gecko-inspired polymeric adhesive materials? In recent years, our understanding of contact electrification mechanisms has developed rapidly with scanning probe microscopy and first-principles calculations [15,16,17]. There are a certain number of studies on the fundament of contact electrification for polymers, and some distinct viewpoints have been raised. For example, the effective work function and tunneling effect are proposed to explain the charge transfer between polymer surfaces [18,19,20]. Meanwhile, Lowell et al. developed the high-energy state theory that electron transfers from high-energy states to lower-energy states achieve an equilibrium state for polymers [21]. A recent study has also confirmed that the interfacial barrier rather than the effective work function difference is the more fundamental parameter to polymer contact electrification [22]. In addition, some researchers believe that the interface contact electrification is related to the difference in the electronic attraction of the surfaces for polymers, rather than the potential barrier [23]. To date, our understanding of the contact electrification mechanism for polymers is far from enough. Hence, a systematic study of the underlying mechanisms of polymer contact electrification based on the electron transfer model is essential for a better understanding of the interfacial adhesion properties of polymer adhesive materials.

Accordingly, the study of contact electrification of gecko setae or polymer adhesives is an essential prerequisite for the research of the mechanisms of biological adhesion and the bio-inspired design of artificial adhesives [24]. Studies on simple adhesion systems (amino acids of geckos’ proteins and monomers of polymeric adhesive materials) are necessary for a thorough understanding of actual adhesion systems. Researchers have found that the prevalent proteins in the gecko setae are mostly glycine-rich or cysteine-rich [25,26,27]. The study of glycine and cysteine interfacial charge transfer would give a deep understanding of contact electrification for geckos. Numerous studies have shown that the contact electrification performance of polymers is also affected by the characteristics of the functional groups and the chemical structure of the polymer. By attaching different functional groups to the surfaces of the polyethylene terephthalate (PET) films, researchers can tune the electron loss or gain ability of the PET surface more easily during contact electrification [28]. Li et al. proposed that the contact electrification on both polymer–polymer and polymer–liquid interfaces can be explained by the electron cloud overlapping and electron-withdrawing ability of functional groups [29]. Thus, studies on the relationships between different functional groups of polymers and contact electrification are necessary for a better understanding of the interfacial effect of gecko toes and gecko-inspired dry adhesives.

In this study, we study the contact electrification performance of amino acids (glycine and cysteine) and monomers of polymeric adhesive materials on the SiO_2_ surface at the atomic scale. SiO_2_ is one of the essential dielectric materials in the natural environment of gecko activities and physical property measurements [30,31,32]. The charge transfer between different functional groups of polymers and the SiO_2_ surface is systematically studied to deeply understand the contact electrification mechanism using the first-principles calculation. The results of this work provide useful theoretical knowledge which is very important for a better understanding of contact electrification and adhesion property of gecko seta and bio-inspired adhesive materials at the SiO_2_ surface, and shed light on the design and application of bio-inspired adhesive materials and structures.

## 2. Methodology

All of the DFT calculations in the present work are carried out by the Vienna Ab initio simulation package (VASP) with the projected augmented wave (PAW) method [33,34,35,36]. The exchange-correlation effects are described by the Perdew–Burke–Ernzerhof (PBE) functional within the generalized gradient approximation [37]. The DFT-D3 method of Grimme is used to correct the effect of van der Waals interactions between the molecule and silicon oxide (quartz) surfaces [38]. For calculations of molecule and surface with a dipole moment, the dipole correction is used. The electronic kinetic energy cutoff of 520 eV is used in the calculations. The k-point mesh of 3 × 3 × 1 Monkhorst–Pack is used in all calculations [39]. The convergence standard is set as follows: the interatomic force is less than 0.01 eV/Å, and the energy change per atom is less than 10^−5^ eV. The vacuum layer in the model is more than 20 Å along the *z*-direction to avoid the mirror self-interaction.

To simulate the interfacial structure, the contact model of the SiO_2_(001) substrate, molecule, and vacuum layer is adopted in this study. The bulk structure of α-SiO_2_ has hexagonal symmetry (P3121). Previous calculations found that the fully hydroxylated SiO_2_ surface is the most stable one [40]. As various types of interface structures can be generated in molecule/SiO_2_ contact models, the O-terminated, OH-terminated, and Si-terminated SiO_2_(001) are all investigated. The O-terminated (Si-terminated) SiO_2_ is modeled by a slab containing five (six) Si atomic layers and twelve O atomic layers. The O atoms at the bottom are passivated by H. The atomic structure of the O-terminated systems is built by the removal of the top surface Si atoms from the Si-terminated systems. The atomic structure of the OH-terminated is constructed by hydroxylating the top surface O atoms from the O-terminated systems. The interfacial properties are calculated using a 3 × 3 SiO_2_(001) supercell. Thus, the O-terminated (Si-terminated) SiO_2_ contains 45 (54) Si atoms, 108 O atoms, and 18 H atoms, and the OH-terminated SiO_2_ contains 45 Si atoms, 108 O atoms, and 36 H atoms (Figure S1). The SiO_2_ surface is first fully relaxed prior to interfacial calculations. For O-terminated or OH-terminated (Si-terminated) SiO_2_ and molecule contact systems, the bottom ten (eleven) atomic layers are fixed in their bulk positions during relax calculations.

**Figure 1:**
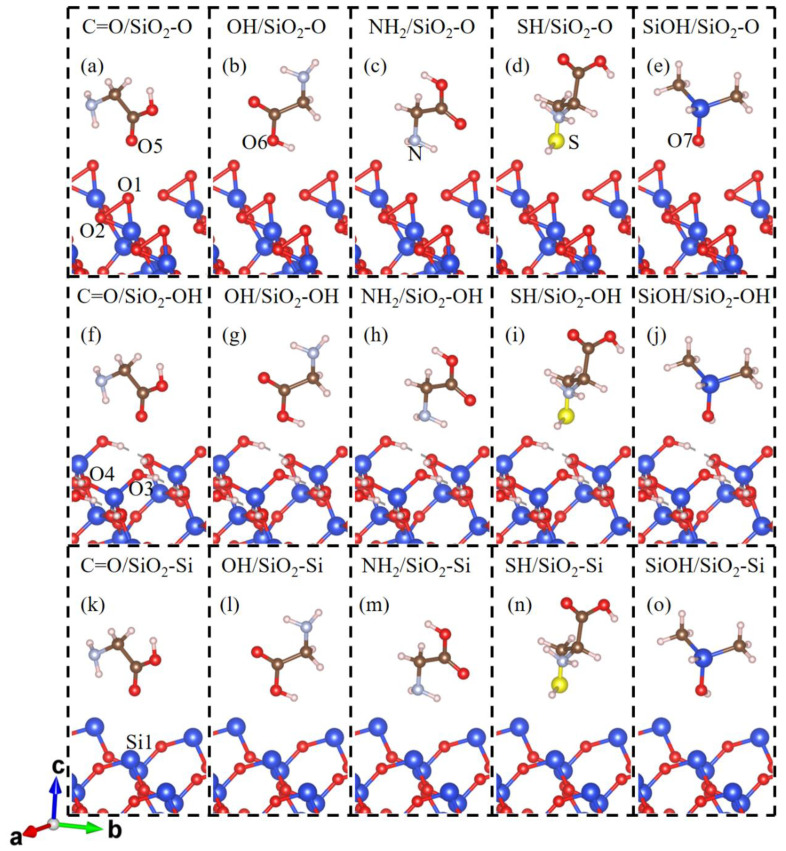
The initial contact conformation for (**a**) C=O group of glycine; (**b**) OH group of glycine; (**c**) NH_2_ group of glycine; (**d**) SH group of cysteine; (**e**) SiOH group of dimethyl siloxane molecule on the O-terminated SiO_2_(001) (SiO_2_-O) surface; (**f**) C=O; (**g**) OH; (**h**) NH_2_; (**i**) SH; (**j**) SiOH groups on the OH-terminated SiO_2_(001) (SiO_2_-OH) surface; (**k**) C=O; (**l**) OH; (**m**) NH_2_; (**n**) SH; and (**o**) SiOH group on the Si-terminated SiO_2_(001) (SiO_2_-Si) surface. The Si, O, H, C, N, and S atoms are drawn in blue, red, white, brown, light blue, and yellow, respectively.

The fact that contact electrification is likely to be related to the molecular structure of the polymer. Hence, in our calculations, the different functional groups of amino acids and monomers of adhesive materials should be considered. A glycine (Gly) molecule has three active sites, carbonyl oxygen (C=O), hydroxyl oxygen (OH), and amino nitrogen (NH_2_) groups. A cysteine (Cys) molecule has the above-mentioned groups and an extra sulfhydryl sulfur (SH) group. In addition to the above functional groups, since the most common polymeric adhesive materials partly differ through their sidechains, we decided to add the silanol group of dimethyl siloxane (C_2_H_8_OSi, DMS), which represents the constituent repeating unit of adhesive polydimethylsiloxane material in the theoretical calculations. Therefore, the most common functional groups of gecko toes and polymer adhesive materials have been considered. The molecule structure is modeled as the Gly, Cys, or DMS molecule in a 14.73 × 12.756 × 40 Å cell, which is the size of the SiO_2_(001) slab for calculations. In calculations, molecules are placed above the top site of the surface atoms of the SiO_2_(001) surface via five related active sites. The molecules have ten or more atoms which involve a number of degrees of freedom in the simulation. Therefore, we have limited our calculations to the models described as the contact atom of SiO_2_ surface (topmost O atom of O-terminated and OH-terminated SiO_2_(001) surface and topmost Si atom of Si-terminated SiO_2_(001) surface) contact atom (O, N or S atom) of the molecule. The corresponding two adjacent atoms (C or Si atom) have the same coordinates in the *x*-axis and *y*-axis to ensure the top site’s relationship, which is kept frozen at the *x*-axis and *y*-axis in the relax calculations. The graphical representations of the initial configurations of molecule-SiO_2_(001) systems are shown in Figure 1.

To quantify the adhesion energy between SiO_2_(001) surface and molecule, *E*_b_ is obtained for various cases of the study:

*E*_b_ = *E* − *E*_sub_ − *E*_mo_, (1)

where *E* is the total energy of the contact systems, and *E*_sub_ (*E*_mo_) is the total energy of the isolated SiO_2_(001) (molecule).

The quantitative estimation of the charge density differences along the *z*-direction is defined as:

Δ*n*(*z*) = *n*(*z*) − *n*_sub_(*z*) − *n*_mo_(*z*), (2)

where *n*(z) is the *xy*-plane-averaged charge density of the systems, and *n*_sub_ (*n*_mo_) is the *xy*-plane-averaged charge density of the isolated SiO_2_ (molecule).

Moreover, the amount of transferred charge is obtained by integrating Δ*n*(*z*) from the interfacial node *z*_0_ (the position of the electrostatic potential peak at the interface) to the vacuum. Thus, the transferred charge *Q* is defined as:

(3) $$Q=-e\int_{z_{0}}^{\infty }\Delta n\left(z\right)dz$$

where −*e* is the charge of an electron, and *z*_0_ is the interfacial point between the SiO_2_ substrate and the molecule, which is discussed below.

## 3. Results and Discussion

The characteristic parameters including interfacial distance, adhesion energy, and the transferred charge of 15 molecule/SiO_2_ configurations are listed in Table 1. The interfacial distances on the SiO_2_-OH surface are slightly larger than that on the SiO_2_-O surface for the molecule with the same group, which are all about 3.0 Å. The equilibrium interfacial atomic distances for the molecule/SiO_2_-Si systems are about 3.6 Å or larger. For most contact systems, the distances are approximately equal to the sums of the corresponding van der Waals atomic radius of interfacial atoms except the SH/SiO_2_-O and SH/SiO_2_-OH systems (~3.0 Å v.s. 3.32 Å), indicating the possible stronger interfacial interaction and charge transfer in the SH/SiO_2_-O and SH/SiO_2_-OH systems.

To quantitatively evaluate the interfacial adhesion, we calculate the adhesion energy of the molecule/SiO_2_ systems (Table 1). Adhesion energy is the result of the interaction of several factors, including hydrogen bonds, van der Waals interaction, and electrostatic interaction. The negative adhesion energy indicates an attraction interaction. The results indicate that the order of adhesion energy for molecule groups binding to the SiO_2_-O or SiO_2_-OH surface is SH > SiOH > NH_2_ > C=O > OH. The SH group of cysteine and SiO_2_-O or SiO_2_-OH contact model has strong adhesion energy. While for the molecule/SiO_2_-Si systems, the order of adhesion energy is SiOH > NH_2_ > SH > OH > C=O, that is, contact with the SiOH group of siloxane facing the SiO_2_-Si surface has strong adhesion energy. When the molecule contacts the SiO_2_-Si surface, the NH_2_ group of glycine has a larger adhesion energy than the SH group of cysteine. Furthermore, when glycine contacts the different SiO_2_ surfaces, the NH_2_ group of glycine has the largest adhesion energy. This indicates that the most possible contact site of the Gly/SiO_2_ system is the amino group of glycine, which agrees with experimental results that glycine is specifically adsorbed on silica surfaces through its ${\mathrm{NH}}_{3}^{+}$ moiety [41]. Thus, for the different molecule/SiO_2_ contact systems, the SH group of cysteine, the SiOH group of siloxane, and the NH_2_ group have a larger adhesion energy, and it can be inferred that SH and SiOH groups may to some extent enhance the interfacial adhesion. Furthermore, the effect of hydrogen bonds on interfacial adhesion energy is also investigated, since the network of hydrogen bonds can reveal the interactional complementarity between the amino acid and specific contact sites for the contact models [42,43]. Considering the electronegativity of interfacial O, N, and S atoms, it is inferred that interfacial H atoms can interact with interfacial O, N, and S atoms in the form of hydrogen. It is clear that molecule/SiO_2_-OH systems can form more hydrogen bonds than molecule/SiO_2_-O systems through the same group, leading to larger adhesion energy than that of most of the molecule/SiO_2_-O systems. For instance, the adhesion energy of the C=O/SiO_2_-O is −0.0568 eV, which is less than that of the C=O/SiO_2_-OH contact system (−0.0888 eV).

**Figure 2:**
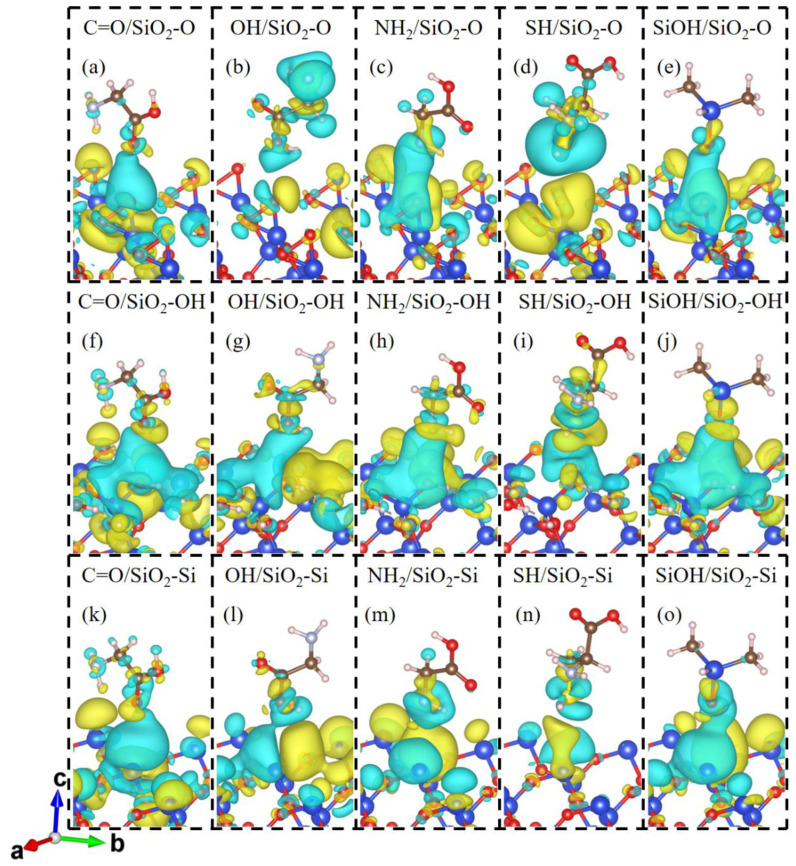
Charge density difference for the contact systems of (**a**) C=O/SiO_2_-O, (**b**) OH/SiO_2_-O, (**c**) NH_2_/SiO_2_-O, (**d**) SH/SiO_2_-O, (**e**) SiOH/SiO_2_-O, (**f**) C=O/SiO_2_-OH, (**g**) OH/SiO_2_-OH, (**h**) NH_2_/SiO_2_-OH, (**i**) SH/SiO_2_-OH, (**j**) SiOH/SiO_2_-OH, (**k**) C=O/SiO_2_-Si, (**l**) OH/SiO_2_-Si, (**m**) NH_2_/SiO_2_-Si, (**n**) SH/SiO_2_-Si, and (**o**) SiOH/SiO_2_-Si. The blue and yellow domains show the electron depleted and accumulated regions, respectively. The value of the iso-surface is set to 2 × 10^−4^ e/bhor^3^ for the OH/SiO_2_-O and SH/SiO_2_-O systems. The value of the iso-surface is set to 8 × 10^−5^ e/bhor^3^ for other contact systems.

To clarify the contact electrification mechanism of gecko toes and bio-inspired adhesive materials, we investigate the charge transfer and charge redistribution at the molecule and SiO_2_ interfaces. The charge density differences in the molecules contacting SiO_2_ surfaces are shown in Figure 2. For molecule and SiO_2_ contact systems, charge accumulations and depletions appear not only in the vicinity of the contacting O or Si atoms of the SiO_2_ substrate but also around the adjacent O or Si atoms, which indicates the delocalization charge distribution between the molecule and SiO_2_ substrate. As displayed in Figure 2a–e, the SiO_2_-O substrate acquires electrons from Gly, Cys, and DMS molecules, forming a charge accumulation region around interfacial O atoms of the SiO_2_ surface and charge depletion regions around the interfacial atoms of molecules. In the molecule/SiO_2_-OH systems (Figure 2f–j), there is significant charge accumulation around the molecule and charge depletion around the surface atoms of the SiO_2_-OH surface, indicating electrons transfer from the SiO_2_ surface to the molecules. Meanwhile, owing to the existence of interfacial hydrogen bonding, the H atom of molecules or SiO_2_ surface and the O, N or S atom of Gly, Cys, or DMS have an interaction with each other and form a charge depletion region at the interface as shown in Figure 2c,e,f–j. For the molecule/SiO_2_-Si systems (Figure 2k–o), charge density differences indicate that the charge on the SiO_2_ surface increases and that on the molecule side decreases when in contact. That is, the electrons transfer from the molecule to the SiO_2_ surface side.

**Figure 3:**
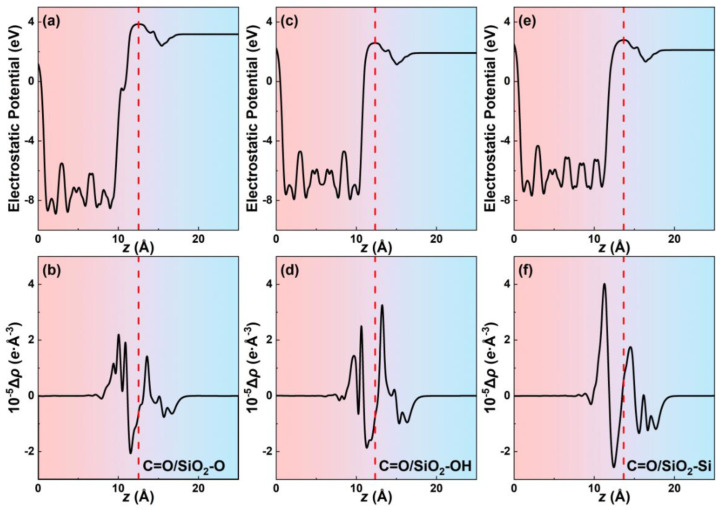
The plane-average electrostatic potentials along the *z* direction of the (**a**) C=O/SiO_2_-O, (**c**) C=O/SiO_2_-OH, and (**e**) C=O/SiO_2_-Si systems. The plane-average charge density differences along the *z* direction of the (**b**) C=O/SiO_2_-O, (**d**) C=O/SiO_2_-OH, and (**f**) C=O/SiO_2_-Si systems.

Quantitative variations of charges at the interface in all contact systems are further calculated using the plane-averaged charge density differences that numerically account for the charge transfer. Figure 3b,d,f exhibit the variation of charge density differences along the *z*-direction for C=O/SiO_2_-O, C=O/SiO_2_-OH, and C=O/SiO_2_-Si contact systems, respectively. The plane-average electrostatic potential is calculated to confirm the interfacial dividing point z_0_ along the *z*-direction of the systems (Figure 3a,c,e). The dividing point z_0_ locates the position of the electrostatic potential peak at the interface, which is marked in the red dotted line in Figure 3. For the C=O/SiO_2_-O and C=O/SiO_2_-Si contact systems, the maximum value of the SiO_2_ side positive peak is larger than that of the glycine positive peak. While for the C=O/SiO_2_-OH systems, the maximum value of the glycine positive peak is larger than that of the SiO_2_ side. To some extent, these results further illustrate that the accumulation of charge density tends to locate at the molecule (SiO_2_) side in the C=O/SiO_2_-OH (C=O/SiO_2_-O and C=O/SiO_2_-Si) contact systems. The plane-averaged charge density differences in other molecule/SiO_2_ contact systems are shown in Figures S2–S5. Thus, for the molecule/SiO_2_-O and molecule/SiO_2_-Si contact systems, the charge in the molecule side tends to decrease and more charge gathers at the SiO_2_ atoms’ top positions, leading to the molecule to SiO_2_ surface charge transfer direction. Meanwhile, for the molecule/SiO_2_-OH contact systems, charge transfers from the SiO_2_ side to the molecule side.

The transferred charge *Q* is calculated as the calculation formula mentioned above, and the corresponding values of *Q* are listed in Table 1. Charge transfers from molecule to SiO_2_ surface when the molecule contacts with the SiO_2_-O or SiO_2_-Si substrates. The molecule/SiO_2_-OH systems have a contrary charge transfer direction. The interfacial hydrogen bonds limit the charge transfer and make the less-transferred charge of the molecule/SiO_2_-OH systems when compared with the molecule/SiO_2_-O systems. Moreover, owing to the unpaired electrons of O atoms, the saturated (OH-terminated) SiO_2_ surfaces have a more limited charge transfer than the O-terminated SiO_2_ surfaces. Under the effect of these two factors, the amounts of transferred charge for the molecule/SiO_2_-O systems are larger than the molecule/SiO_2_-OH systems. The value of *Q* is in the order of SH > OH > NH_2_ > SiOH > C=O for the molecule/SiO_2_-O systems. The sequence of *Q* for the molecule/SiO_2_-OH systems is SH > OH > SiOH > NH_2_ > C=O. Thus, the transferred charge *Q* of the SH/SiO_2_-O and OH/SiO_2_-O (SH/SiO_2_-OH and OH/SiO_2_-OH) contact systems is larger than other molecule/SiO_2_-O (molecule/SiO_2_-OH) systems. The transferred charge *Q* of different molecule/SiO_2_-Si systems is in an order of NH_2_ > C=O > SiOH > OH > SH, that is, the NH_2_/SiO_2_-Si system has the largest transferred charge in all molecule/SiO_2_-Si systems. The transferred charges of the SH/SiO_2_-O and SH/SiO_2_-OH systems are −0.09641 *e* and 0.00798 *e*, respectively, which are much larger than that of other molecule/SiO_2_-O and molecule/SiO_2_-OH systems. The difference between the electronegativity of the interfacial O atom and the S atom may affect the charge transfer. Meanwhile, combined with the hydrogen bond and van der Waals interaction, the interfacial charge transfer affects the interfacial interactions and finally affects the interfacial adhesion energy.

**Figure 4:**
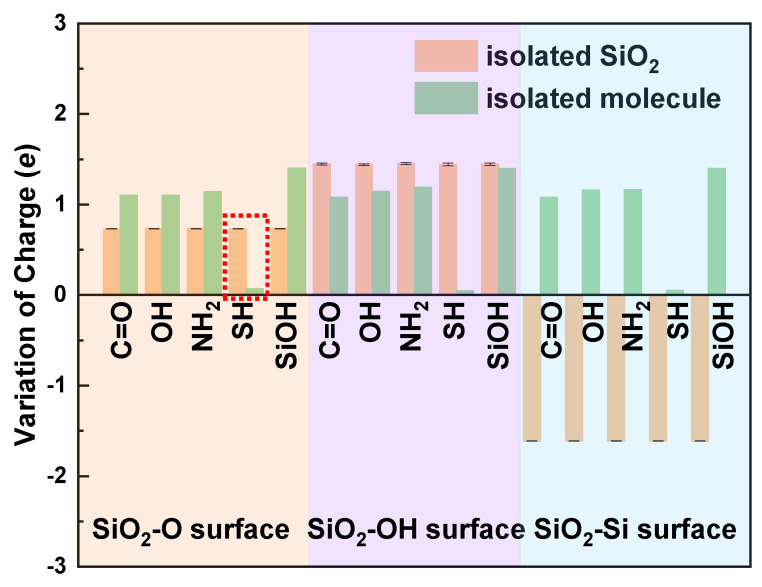
Bader charge analysis on atoms at the isolated SiO_2_ surface and the molecule.

To explore the determinant of the direction of interfacial charge transfer, we carry out the Bader Charge Analysis of the single contact atom in the free molecule and the isolated SiO_2_ surfaces (Figure 4). When in contact, charge trends transfer from the surface with more charges to the surface with fewer charges. Thus, charge transfers from the molecule to the SiO_2_-O and SiO_2_-Si surface after contact (see light orange and light cyan region in Figure 4), and charge tends to transfer from the SiO_2_-OH surface to the molecule side (see the light magenta region in Figure 4) to balance the interface charge. However, for the SH/SiO_2_-O contact system (see the red dotted box in Figure 4), the charge transfer direction cannot be directly obtained by comparing the amounts of the variation of charge for the contact atoms. We may think that charge transfers from the SiO_2_-O surface to the cysteine side (Figure 4). However, the charge transfer direction is from molecule to SiO_2_ surface for the SH/SiO_2_-O system. Thus, the charge transfer mechanism of the SH/SiO_2_-O system needs to be further investigated. The charge transfer behavior of the SH/SiO_2_-O system is considered by detailed Bader Charge Analysis. The O1 atom in the isolated SiO_2_ obtains 0.730997 *e*, which is smaller than that of the contact system (0.782837 *e*). The S atom in the isolated cysteine obtains a larger charge than that of the system (0.067708 vs. −0.013996 *e*). The changes in Bader charge for contact (O1 and S) atoms further prove that charge transfers from cysteine to the SiO_2_-O surface. These things considered, we tried to find the effect of interfacial structure on charge transfer. The length of the O1 and O2 bond on the SiO_2_-O surface is 1.72 Å, which is larger than the bond length of O1–O2 (1.64 Å) for SiO_2_-O substrate in other systems, indicating the weaker interaction between the interfacial O atoms. The interaction between the O1 atom and S atom of cysteine strengthens, and charge trends transfer from the larger radius S atom to the smaller radius O atom, leading to the increasing charge transfer at the interface. Thus, charge transfers from the cysteine molecule to the SiO_2_-O surface, which agrees with other molecule/SiO_2_ contact systems.

**Figure 5:**
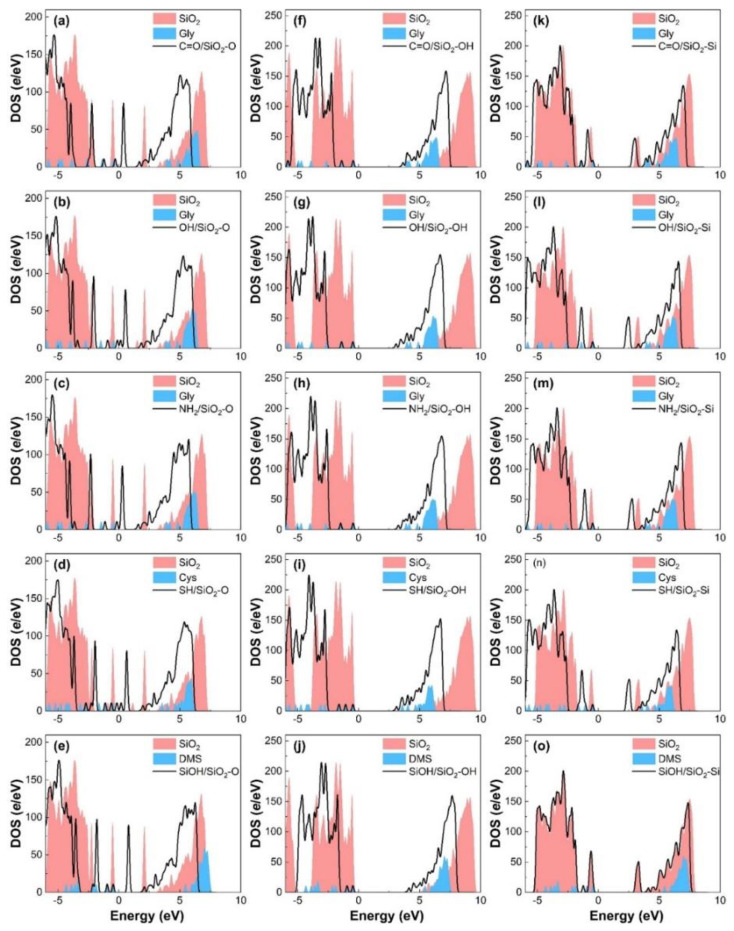
DOS of SiO_2_-O substrate with (black solid line) and without (red shadow) molecules’ (**a**) C=O, (**b**) OH, (**c**) NH_2_, (**d**) SH, and (**e**) SiOH group contact. DOS of SiO_2_-OH substrate with (black solid line) and without (red shadow) molecules’ (**f**) C=O, (**g**) OH, (**h**) NH_2_, (**i**) SH, and (**j**) SiOH group contact. DOS of SiO_2_-Si substrate with (black solid line) and without (red shadow) molecules’ (**k**) C=O, (**l**) OH, (**m**) NH_2_, (**n**) SH, and (**o**) SiOH group contact. The blue shadow denotes the DOS of the corresponding isolated molecule. The Fermi level is set to zero.

To gain insight into the nature of contact electrification between molecules and different SiO_2_ surfaces, we calculate the total density of states (TDOS) of these contact systems. Figure 5 shows that the contact between the molecule and SiO_2_ substrate causes the states to shift toward the lower energy levels after contact, indicating the stabilization of contact systems. For the molecule/SiO_2_-O and molecule/SiO_2_-Si contact systems, the contribution of molecular contact and charge transfer to electronic orbit can change the minimum of the conduction band of contact systems, which narrows the bandgap of the contact system. Similarly, for the molecule/SiO_2_-OH contact systems, the impurity state induced by the molecule is located below the conduction band, which causes the band gap of the contact system to become narrower after contact. To further explore the direction of charge transfer and contact electrification mechanism of molecule/SiO_2_ contact systems, the high-energy states originated by the TDOS of the isolated SiO_2_ substrate and the molecule are considered. The high-energy state electrons above the Fermi level influence the charge transfer and the difference in the high-energy state electrons (first peak of the density of states energy) of isolated surfaces can be used to further confirm the direction of charge transfer. The first peak of isolated SiO_2_-O substrate density of states energy (DOS) above the Fermi level in the energy range of [0.9 eV, 2.4 eV], and the first peak of free molecule DOS above the Fermi level in the energy range of [3.7 eV, 4.3 eV] or [5.3 eV, 5.6eV] (Figure 5a–e). The energy of high-energy states for the free molecule is larger than that of the isolated SiO2-O slab. Thus, higher energy state electrons of molecules tend to transfer to the SiO_2_-O substrate. For the molecule/SiO_2_-Si system (Figure 5k–o), the first peak of isolated SiO_2_-Si substrate and free molecule DOS above the Fermi level is in the range of [3.0 eV, 3.4 eV] and [3.4 eV, 4.0 eV] (or [5.3 eV, 5.6 eV]). We can find the same energy states magnitude distribution of isolated surfaces above the Fermi energy, meaning the same interfacial charge transfer direction (from molecule to SiO_2_-Si substrate). As shown in Figure 5f–j, the energy of the first peak above the Fermi energy for the free molecule is less than that of isolated SiO_2_-OH slabs, causing charge transfers from the SiO_2_-OH surface to glycine, which leads to the reversed charge transfer direction from molecule/SiO_2_-O and molecule/SiO_2_-Si contact systems. Thus, the direction of interfacial charge transfer is further confirmed by the energy difference in the high-energy state electrons above the Fermi level.

**Figure 6:**
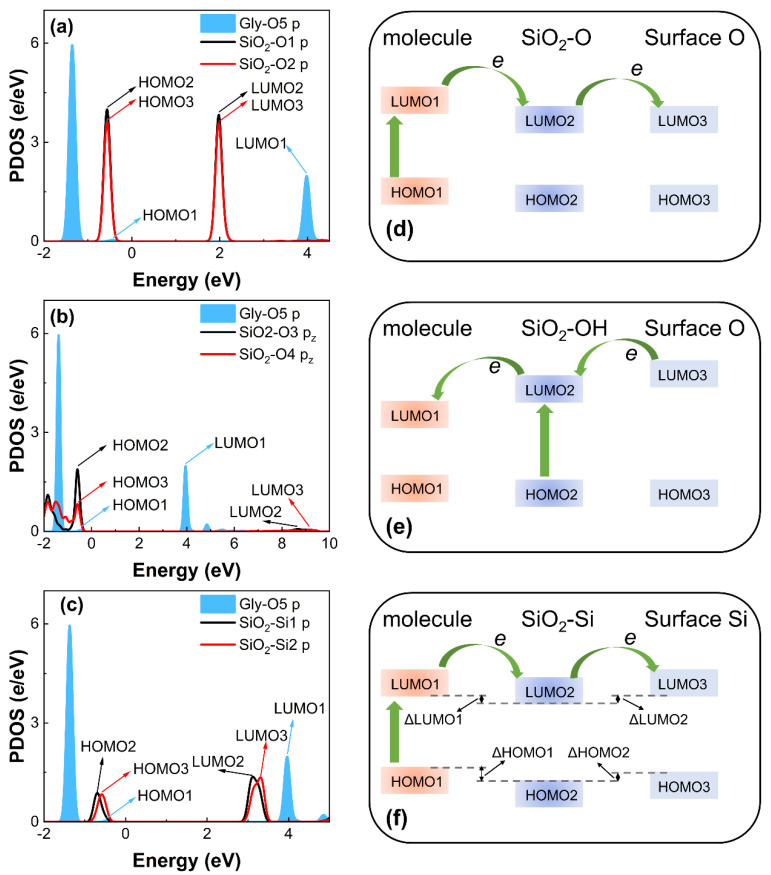
The PDOS electrons of the contact and neighbor atoms in (**a**) Gly and SiO_2_-O surface, (**b**) Gly and SiO_2_-OH surface, and (**c**) Gly and SiO_2_-Si surface before contact. The black curve shows the PDOS of contact O1, O3, and Si1 atoms in SiO_2_ surface. The red curve shows the PDOS of neighbor O2, O4, and Si2 (adjacent Si atom) atoms in SiO_2_ surface. The blue shadow denotes the PDOS of corresponding contact O5, O6, N, S and O7 atoms in isolated molecule. Fermi level is set to zero. Schematic diagrams of charge transfer mechanism of (**d**) molecule/SiO_2_-O, (**e**) molecule/SiO_2_-OH, and (**f**) molecule/SiO_2_-Si contact systems.

In order to understand the contact electrification property and the mechanism of the charge transfer at the interface, the electronic energy level for the considered complexes is analyzed. The valence band maximum (VBM) and conduction band minimum (CBM) of SiO_2_ surface atoms are calculated to describe the electronic energy. Their equivalent in a molecule is the energies of the highest occupied molecular orbital (HOMO) and the lowest unoccupied molecular orbital (LUMO), respectively. These parameters are vital ingredients that define the electronic structure and control processes of charge transfer across interfaces. The HOMO and LUMO of the molecular contact atom are marked as HOMO1 and LUMO1, and the VBM and CBM of the SiO_2_ contact atom are marked as HOMO2 and LUMO2, which are marked in Figure 6a–c and Figure S6, and all included in Table S1. The energy of the LUMO is partially occupied and is used to accept the transferable electrons. The values of LUMO1 and LUMO2 are different; when the systems contact, electrons transfer from the larger LUMO to the smaller LUMO. When the molecule contacts the SiO_2_-Si surface, electron transition will first happen from HOMO1 to LUMO1 in the molecule itself, then electrons will transfer from the LUMO1 of the molecule to LUMO2 of the SiO_2_, making the interface more energetically stable (Figure 6f). As shown in Figure 2, charge accumulations or depletions appear around the adjacent atoms of the SiO_2_ substrate, not just at the two contacting atoms. The HOMO3 and LUMO3 of the SiO_2_ interfacial neighboring atom should also be considered. Thus, electrons further transfer from LUMO2 to LUMO3. Meanwhile, LUMO3 is larger than LUMO2, indicating the hindrance of interfacial charge transfer and reducing the amount of transferred charge. The electron transition mechanism of the molecule/SiO_2_-O and molecule/SiO_2_-OH systems are similar to the molecule/SiO_2_-Si systems. For the molecule/SiO_2_-O systems (Figure 6d), the value of LUMO1 of the molecule is larger than the LUMO2 of SiO_2_. So, when contacting each other, electrons first transfer from HOMO1 to LUMO1 in the molecule itself, and then electrons transfer from LUMO1 to LUMO2 between interfaces. Furthermore, the value of LUMO2 is equal to LUMO3, which means that obtained charge is easy to transfer from LUMO2 to LUMO3 at the SiO_2_ surface leading to the non-local charge distribution, as shown in Figure 2a. For the molecule/SiO_2_-OH system (Figure 6e), the value of LUMO1 is less than LUMO2. Electrons transfer from HOMO2 to LUMO2 in the SiO_2_ itself, and then electrons transfer from LUMO2 to LUMO1. Meanwhile, LUMO3 is larger than LUMO2, which represents that charge will transfer from adjacent atoms to the contact atoms owing to the electron depletion of the contact O atom on the SiO_2_ surface.

Table 1 (ΔHOMO1) is defined as ΔLUMO1 = LUMO1 − LUMO2 (ΔHOMO1 = HOMO1 − HOMO2) and specific data are included in Table S2. The energy gap and energy levels are central to the definition of carrier injection and extraction at the interfaces [44]. Thus, in this paper, the energy gaps ΔLUMO1 and ΔHOMO1 reflect the charge transfer direction and the ability to attract electrons of the two contact atoms, respectively. As mentioned above, charge accumulations or depletions appear around the SiO_2_ substrate after contact. The energy gap between the contact atom and the adjacent atom in the SiO_2_ surface should also be considered. Then, the energy gap ΔLUMO2 (ΔHOMO2) is defined as ΔLUMO2 = LUMO2 − LUMO3 (ΔHOMO2 = HOMO2 − HOMO3) to further elaborate the effect of SiO_2_ surface charge distribution to interfacial charge transfer and the electronic attraction. Thus, the relation between transferred charge *q* and the energy gaps (ΔLUMO1, ΔHOMO1, ΔLUMO2, and ΔHOMO2) can be established by considering a correlation analysis.

A multiple linear regression (MLR) model is used to model the charge transfer in terms of parameters of ΔLUMO1, ΔHOMO1, ΔLUMO2, and ΔHOMO2. If *Q* > 0, charge transfer *Q* has a negative association with the response variable ln(|*Q*|), if *Q* < 0, *Q* is proportional to ln(|*Q*|). *Q* and ln(|*Q*|) do not exhibit monotonic behavior. So, the positive and negative sign for the below MLR equation (for monotonicity of *Q* and ln(|*Q*|)) is used for the mathematical meaning rather than the physical meaning.

The obtained MLR equation is as follow:

ln(|*Q*|) = ±(−6.11 + 0.23ΔLUMO1 + 5.08ΔHOMO1 − 28.22ΔLUMO2 + 55.11ΔHOMO2), (4)

The coefficient of determination (R^2^) for the prediction capability of the MLR model is obtained as 95.39%. ΔLUMO2 has a negative effect on charge transfer, indicating the hindrance of charge transfer or the relatively localized charge distribution, while ΔLUMO1, ΔHOMO1, and ΔHOMO2 have a positive effect. ΔLUMO1 determines the direction of charge transfer and, even more, affects the interfacial charge interaction. The coefficients of ΔLUMO1 and ΔHOMO1 are equal to 0.23 and 5.08, respectively. Meanwhile, the value of ΔLUMO1 is nearly an order of magnitude larger than ΔHOMO1 for the same contact system (Table S2), which indicates that the coefficients of independent variables ΔLUMO1 and ΔHOMO1 have a similar positive effect on charge transfer. For some contact systems, ΔLUMO2 and ΔHOMO2 are very small negative numbers, and then the relatively large value of the coefficients will hinder the charge transfer and the redistribution of electrons on the SiO_2_ surface. Therefore, we can obtain a quantitative relation between the transferred charge and energy gap to evaluate the contact electrification of polymers. This demonstrates that when molecules (Gly, Cys, and DMS) come into contact with SiO_2_, ΔLUMO1 determines the charge transfer direction at the interface, ΔLUMO2 affects the charge transfer at the SiO_2_, and ΔHOMO1 and ΔHOMO2 show that the charge transfer in contact electrification is closely related to the electron attraction from the contact surfaces and atoms.

## 4. Conclusions

In this paper, the electronic behaviors and charge transfer at the contact interfaces of molecule/SiO_2_ systems are studied using density functional theory calculations. The different contact configurations of molecule groups and SiO_2_ surfaces are analyzed. When the molecule contacts the SiO_2_-O and SiO_2_-OH surfaces through the sulfhydryl group, stronger adhesion energy is obtained in comparison to carbonyl, hydroxyl, amino, and silanol groups. The SiOH/SiO_2_-Si contact model has stronger adhesion energy than other molecule/SiO_2_-Si systems. Furthermore, for the different Gly/SiO_2_ systems, the NH_2_ group of glycine has the largest adhesion energy, respectively. Charge transfers from molecule to SiO_2_-O and SiO_2_-Si surfaces. Meanwhile, the charge transfer direction reverses for the molecule/SiO_2_-OH contact systems. The sulfhydryl or hydroxyl groups of the molecule contacting the SiO_2_-OH and SiO_2_-OH surfaces have the larger transferred charge. For the molecule/SiO_2_-Si systems, the NH_2_/SiO_2_-Si system has the largest transferred charge. The direction of the transferred charge is determined by the Bader charge of the isolated contact atoms. In addition, charge tends to transfer from the surface with more charge to the surface with less charge. The charge transfer direction can be further demonstrated by the energy difference in the high-energy state electrons of the molecule and the SiO_2_ surface above the Fermi level. Further investigation indicates that electrons transfer from the HOMO to the LUMO of the molecule or SiO_2_ itself, then electrons transfer from the higher LUMO to the lower LUMO, and finally, electrons transfer between the LUMO2 and LUMO3 of atoms in SiO_2_ surface, making the interface more energetically stable after contact. The energy gap represented by ΔLUMO1 and ΔLUMO2 signifies the charge transfer direction. ΔHOMO1 and ΔHOMO2 reflect the electronic attraction and interfacial charge transfer. Thus, the amount of the transferred charge can be related to the energy gaps (ΔLUMO1, ΔHOMO1, ΔLUMO2, and ΔHOMO2) between the contacting surfaces, and then charge transfer *Q* can be evaluated by the quantitative relationship of these four parameters to predict the interfacial charge transfer behavior. Thus, this study provides a more accurate method to estimate the relations between charge transfer and the variations of energy states for the polymers’ contact electrification. Insights into the interaction between Gly, Cys, or DMS and SiO_2_ may therefore give some important guidance to the further study of contact electrification and adhesion modulation of gecko and bioinspired dry adhesives.

## Figures and Tables

**Table 1:** The interfacial distances *d*_eq_, adhesion energy *E*_b_, and charge transfer *q* of molecules Gly, Cys, and DMS contacted with SiO_2_ slab, respectively. Positive (negative) *q* represents that charge transfers from SiO_2_ (molecule) to molecule (SiO_2_) surface.

	C=O	OH	NH_2_	SH	SiOH
O-terminated SiO_2_ surface					
*d*_eq_ (Å)	3.02	3.032	3.058	2.979	3.025
*E*_b_ (eV)	−0.0568	−0.0558	−0.0598	−0.1747	−0.0723
*q* (*e*)	−0.00162	−0.05576	−0.01261	−0.09641	−0.00242
OH-terminated SiO_2_ surface					
*d*_eq_ (Å)	3.056	3.047	3.092	3.069	3.064
*E*_b_ (eV)	−0.0888	−0.0885	−0.1022	−0.136	−0.1061
*q* (*e*)	0.00036	0.00232	0.00204	0.00798	0.00229
Si-terminated SiO_2_ surface					
*d*_eq_ (Å)	3.607	3.611	3.652	3.877	3.584
*E*_b_ (eV)	−0.0791	−0.095	−0.1088	−0.1079	−0.1161
*q* (*e*)	−0.00234	−0.00098	−0.00769	−0.00045	−0.00155

## Data Availability

The computational data on the results reported in this manuscript are available upon official request to the corresponding authors.

## Supplementary Materials

The following supporting information can be downloaded at: https://www.mdpi.com/article/10.3390/biomimetics7040216/s1. Figure S1. The initial conformation of the isolated (a) SiO_2_-O, (b) SiO_2_-OH, (c) SiO_2_-Si surface, respectively; Figure S2. The plane-average electrostatic potentials along the *z* direction of the (a) OH/SiO_2_-O, (c) OH/SiO_2_-OH, (e) OH/SiO_2_-Si systems. The plane-average charge density differences along the *z* direction of the (b) OH/SiO_2_-O, (d) OH/SiO_2_-OH, (f) OH/SiO_2_-Si systems; Figure S3. The plane-average electrostatic potentials along the *z* direction of the (a) NH_2_/SiO_2_-O, (c) NH_2_/SiO_2_-OH, (e) NH_2_/SiO_2_-Si systems. The plane-average charge density differences along the *z* direction of the (b) NH_2_/SiO_2_-O, (d) NH_2_/SiO_2_-OH, (f) NH_2_/SiO_2_-Si systems; Figure S4. The plane-average electrostatic potentials along the *z* direction of the (a) SH/SiO_2_-O, (c) SH/SiO_2_-OH, (e) SH/SiO_2_-Si systems. The plane-average charge density differences along the *z* direction of the (b) SH/SiO_2_-O, (d) SH/SiO_2_-OH, (f) SH/SiO_2_-Si systems; Figure S5. The plane-average electrostatic potentials along the *z* direction of the (a) SiOH/SiO_2_-O, (c) SiOH/SiO_2_-OH, (e) SiOH/SiO_2_-Si systems. The plane-average charge density differences along the *z* direction of the (b) SiOH/SiO_2_-O, (d) SiOH/SiO_2_-OH, (f) SiOH/SiO_2_-Si systems; Figure S6. The PDOS electrons of the contact and neighbor atoms in (a) Gly-OH and SiO_2_-O surface, (b) Gly-NH_2_ and SiO_2_-O surface, (c) Cys-SH and SiO_2_-O surface, (d) DMS-SiOH and SiO_2_-O surface, (e) Gly-OH and SiO_2_-OH surface, (f) Gly-NH_2_ and SiO_2_-OH surface, (g) Cys-SH and SiO_2_-OH surface, (h) DMS-SiOH and SiO_2_-OH surface, (i) Gly-OH and SiO_2_-Si surface, (j) Gly-NH_2_ and SiO_2_-Si surface, (k) Cys-SH and SiO_2_-Si surface, and (l) DMS-SiOH and SiO_2_-Si surface, before contact. The black curve shows the PDOS of contact O1, O3, and Si1 atoms in SiO_2_ surface, respectively. The red curve shows the PDOS of neighbor O2, O4, and Si2 (adjacent Si atom) atoms in SiO_2_ surface, respectively. The blue shadow denotes the PDOS of corresponding contact O5, O6, N, S and O7 atoms in isolated molecule, respectively. Fermi level is set to zero; Table S1. High energy state of molecule’s contact atom LUMO1, low energy state of molecule’s contact atom HOMO1, high energy state of contact atom in SiO_2_ slab LUMO2, low energy state of contact atom in SiO_2_ slab HOMO2, high energy state of the adjacent atom to SiO_2_ interfacial atom LUMO3, low energy state of the adjacent atom to SiO_2_ interfacial atom HOMO3; Table S2. ΔLUMO1 is the difference of the high energy state between the molecule contact atom and SiO_2_ contact atom, which is defined as ΔLUMO1=LUMO1-LUMO2. ΔHOMO1 is the difference of the low energy state between the molecule contact atom and SiO_2_ contact atom, which is defined as ΔHOMO1=HOMO1-HOMO2. ΔLUMO2 is the difference of the high energy state between the SiO2 contact atom and the adjacent atom, which is defined as ΔLUMO2=LUMO2-LUMO3. ΔHOMO2 is the difference of the low energy state between the SiO2 contact atom and the adjacent atom, which is defined as ΔHOMO2=HOMO2-HOMO3. *q* is the transferred charge between the interface. ln|*q*| is the natural logarithm of the absolute values of *q*. The sign of the ln|*q*| is related its physical meaning and ±ln|*q*| is negatively correlated with *q*.
