# Supplementary material for: Contact Electrification of Biological and Bio-Inspired Adhesive Materials on SiO2 Surfaces: Perspectives from DFT Calculations
Source: Biomimetics (Basel). 2022 Nov 28;7(4):216. doi: 10.3390/biomimetics7040216 (PMC9775756; doi:10.3390/biomimetics7040216)
Supplement: Supplementary file 1 [file biomimetics-07-00216-s001.zip › biomimetics-1974230-supplementary.pdf]

Contact Electrification of Biological and Bio-Inspired Adhesive Materials on SiO_2 Surfaces: Perspectives from DFT Calculations

Jing Tao[1,2], Linfeng Wang[1*], Kaixuan Kong[1], Minhao Hu[1], Zhendong Dai[1]

[1] Jiangsu Provincial Key Laboratory of Bionic Functional Materials, College of Mechanical and Electrical Engineering, Nanjing University of Aeronautics and Astronautics, Nanjing 210016, China

[2] College of Aerospace Engineering, Nanjing University of Aeronautics and Astronautics, Nanjing 210016, China

* Correspondence: wanglf@nuaa.edu.cn

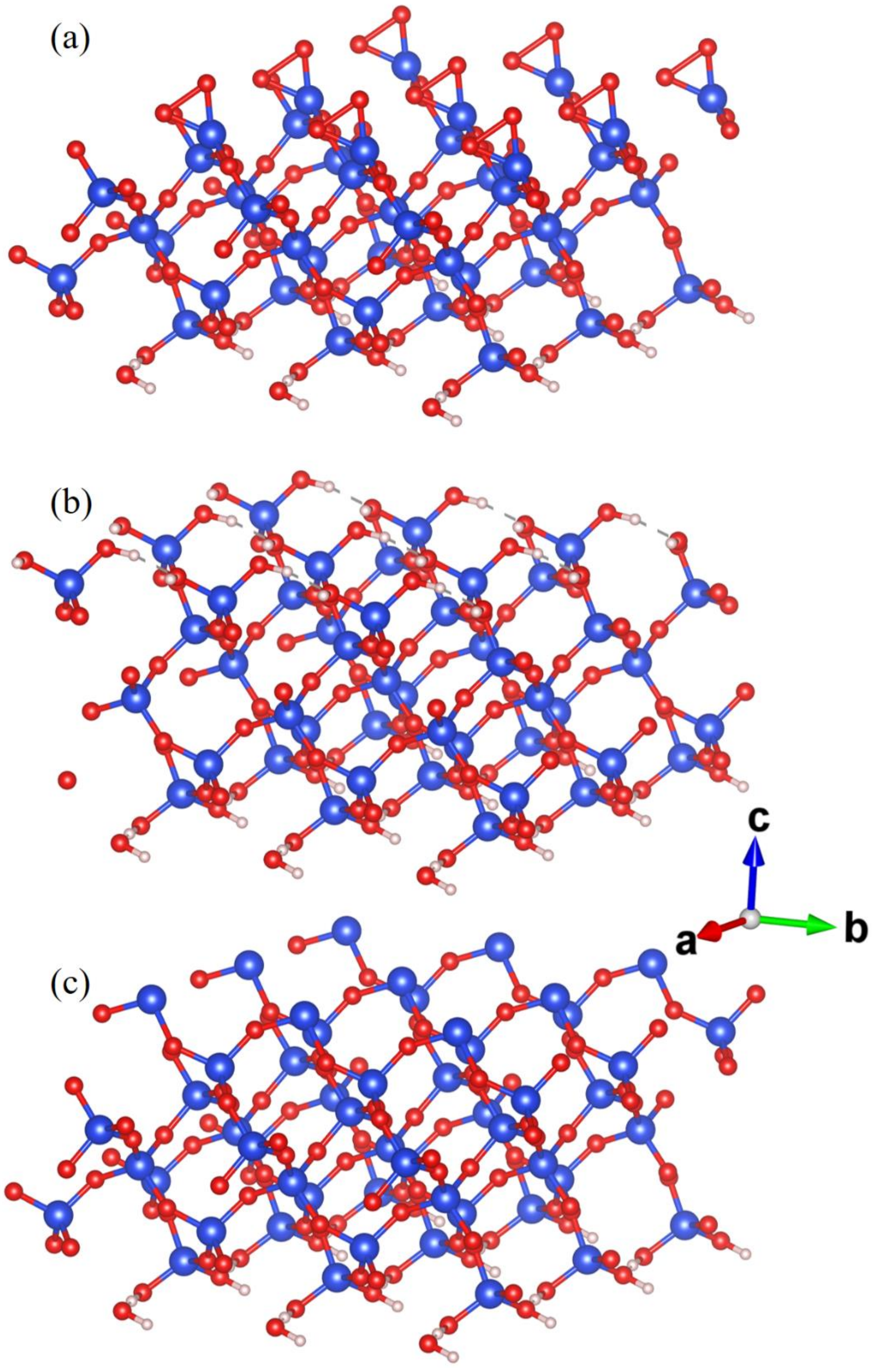

Figure S1. The initial conformation of the isolated (**a**) SiO_2-O, (**b**) SiO_2-OH, (**c**) SiO_2-Si surface, respectively.

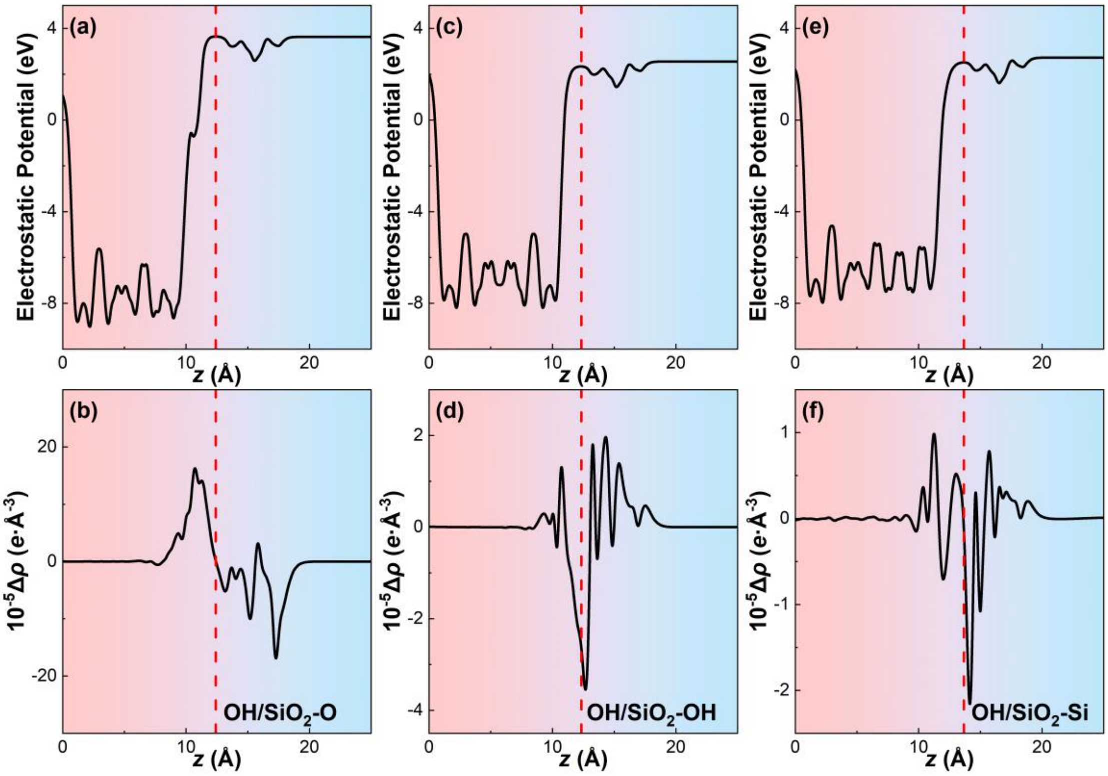

Figure S2. The plane-average electrostatic potentials along the *z* direction of the (**a**) OH/SiO_2-O, (**c**) OH/SiO_2-OH, (**e**) OH/SiO_2-Si systems. The plane-average charge density differences along the *z* direction of the (**b**) OH/SiO_2-O, (**d**) OH/SiO_2-OH, (**f**) OH/SiO_2-Si systems.

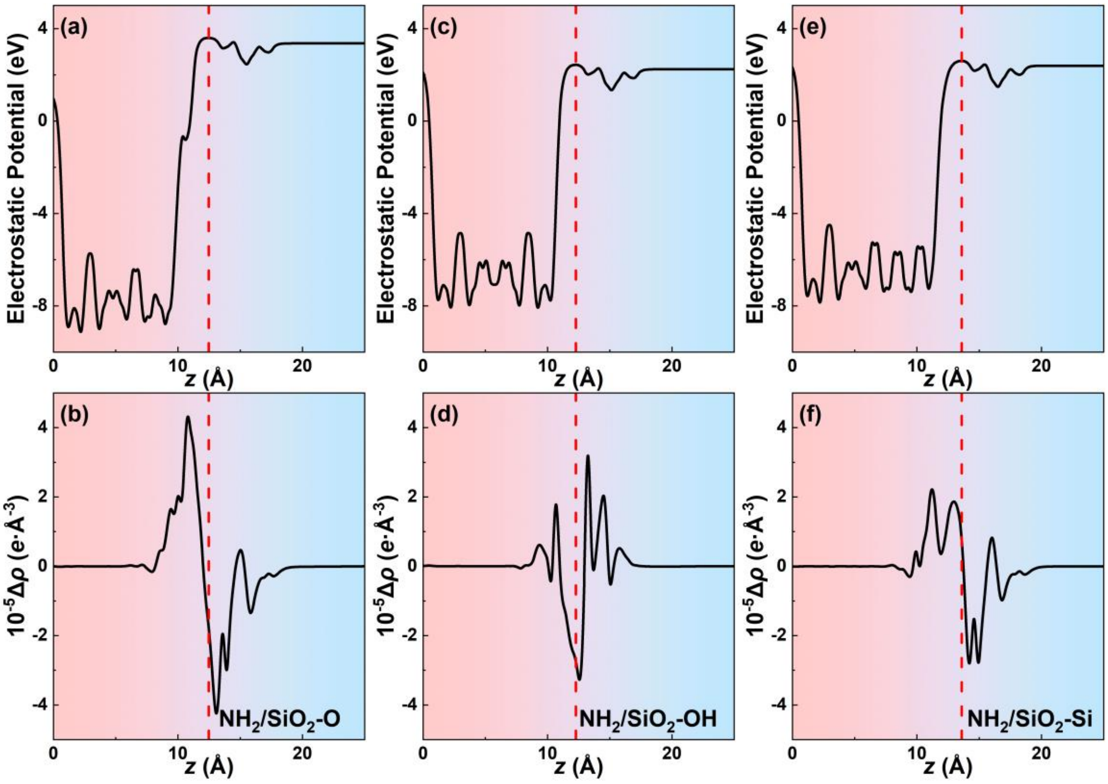

Figure S3. The plane-average electrostatic potentials along the *z* direction of the (**a**) NH_2/SiO_2-O,

(**c**) NH_2/SiO_2-OH, (**e**) NH_2/SiO_2-Si systems. The plane-average charge density differences along the *z* direction of the (**b**) NH_2/SiO_2-O, (**d**) NH_2/SiO_2-OH, (**f**) NH_2/SiO_2-Si systems.

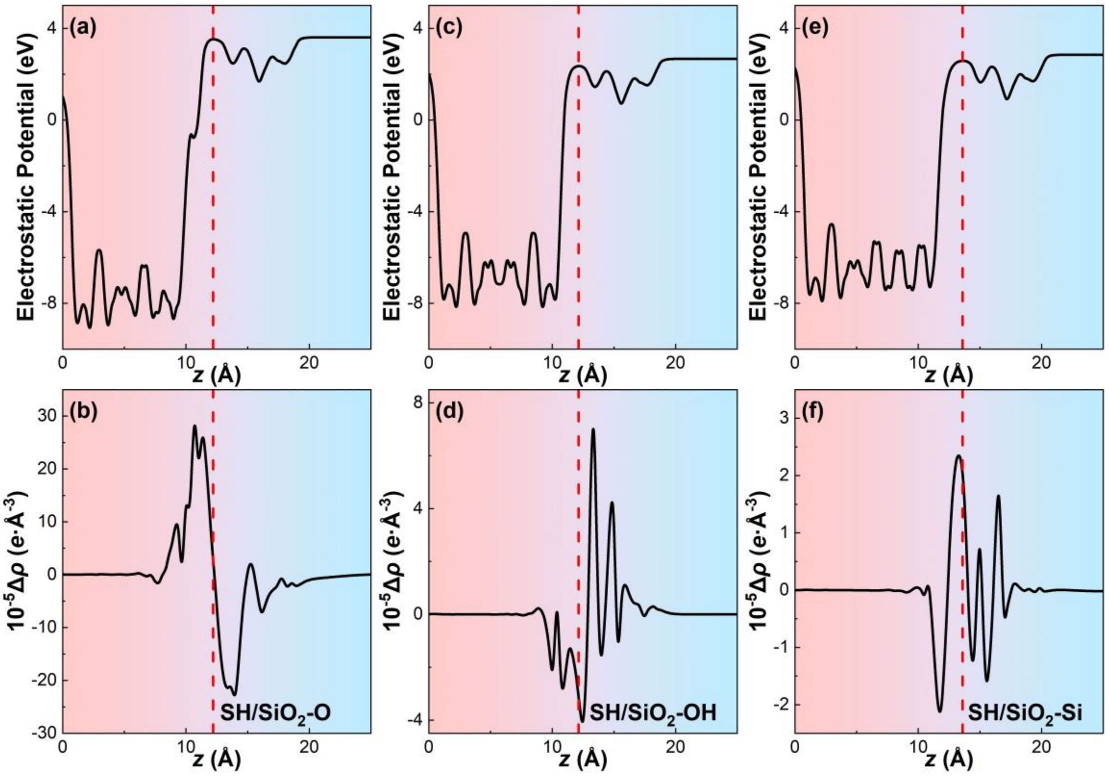

Figure S4. The plane-average electrostatic potentials along the *z* direction of the (**a**) SH/SiO_2-O, (**c**) SH/SiO_2-OH, (**e**) SH/SiO_2-Si systems. The plane-average charge density differences along the *z* direction of the (**b**) SH/SiO_2-O, (**d**) SH/SiO_2-OH, (**f**) SH/SiO_2-Si systems.

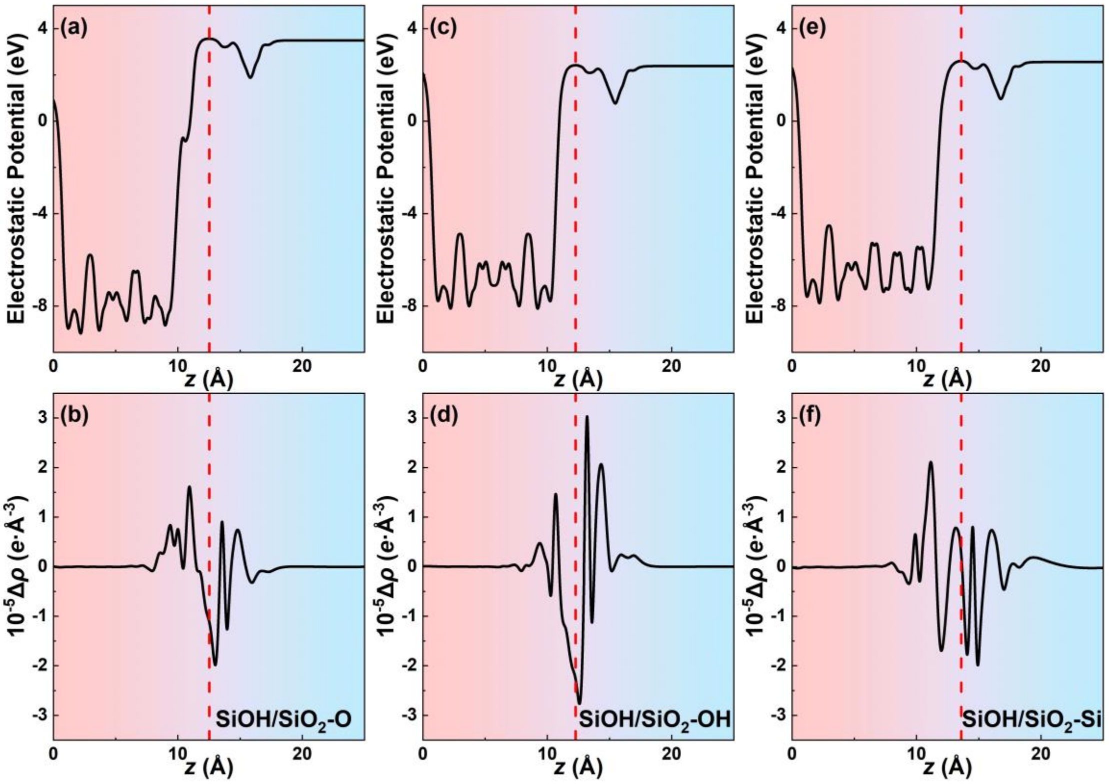

Figure S5. The plane-average electrostatic potentials along the *z* direction of the (**a**) SiOH/SiO_2-O, (**c**) SiOH/SiO_2-OH, (**e**) SiOH/SiO_2-Si systems. The plane-average charge density differences along the *z* direction of the (**b**) SiOH/SiO_2-O, (**d**) SiOH/SiO_2-OH, (**f**) SiOH/SiO_2-Si systems.

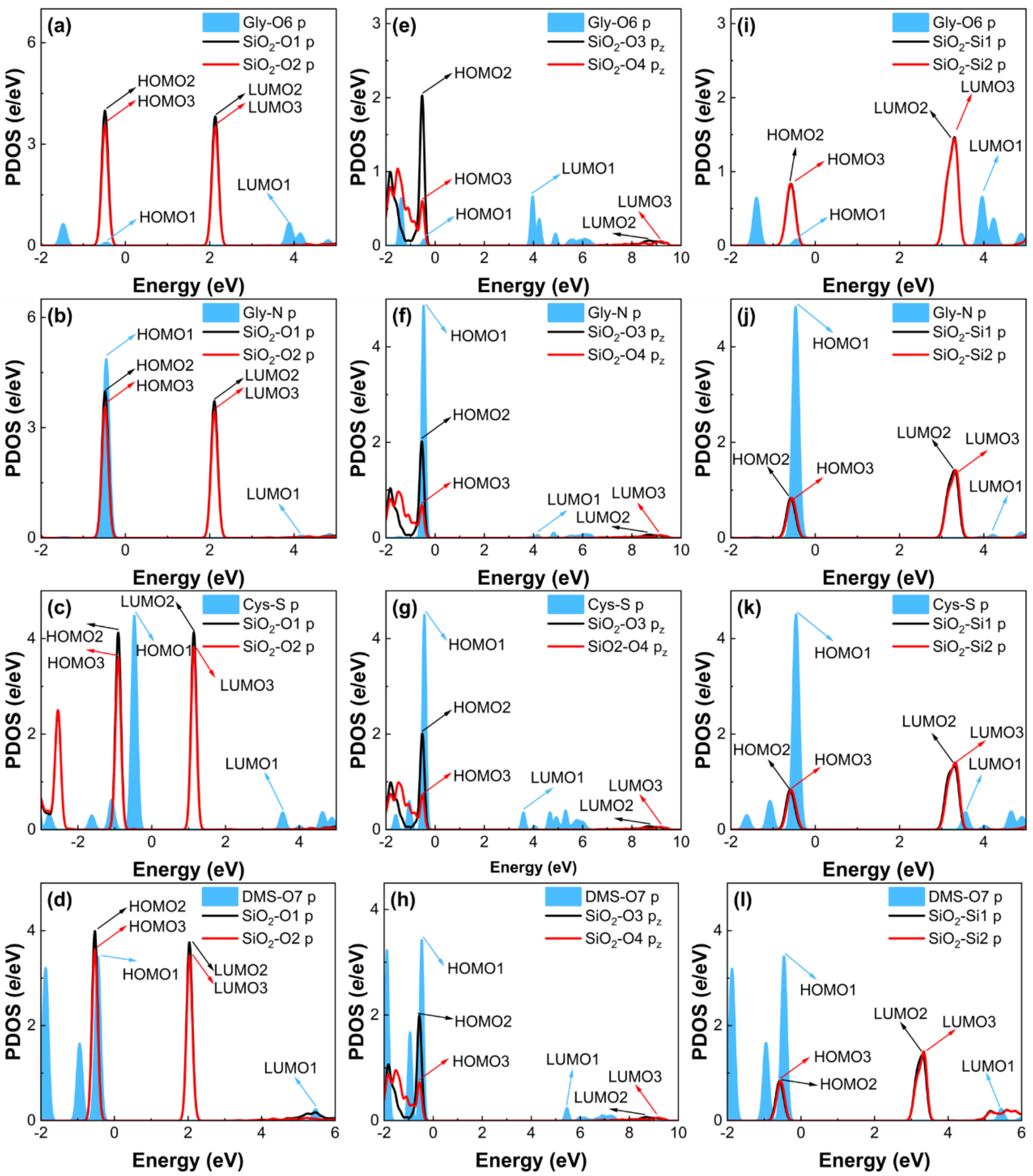

Figure S6. The PDOS electrons of the contact and neighbor atoms in (**a**) Gly-OH and SiO_2-O surface, (**b**) Gly-NH_2 and SiO_2-O surface, (**c**) Cys-SH and SiO_2-O surface, (**d**) DMS-SiOH and SiO_2-O surface, (**e**) Gly-OH and SiO_2-OH surface, (**f**) Gly-NH_2 and SiO_2-OH surface, (**g**) Cys-SH and SiO_2-OH surface, (**h**) DMS-SiOH and SiO_2-OH surface, (**i**) Gly-OH and SiO_2-Si surface, (**j**) Gly-NH_2 and SiO_2-Si surface, (**k**) Cys-SH and SiO_2-Si surface, and (**l**) DMS-SiOH and SiO_2-Si surface, before contact. The black curve shows the PDOS of contact O1, O3, and Si1 atoms in SiO_2 surface, respectively. The red curve shows the PDOS of neighbor O2, O4, and Si2 (adjacent Si atom) atoms in SiO_2 surface, respectively. The blue shadow denotes the PDOS of corresponding contact O5, O6, N, S and O7 atoms in isolated molecule, respectively. Fermi level is set to zero.

Table S1. High energy state of molecule's contact atom LUMO1, low energy state of molecule's contact atom HOMO1, high energy state of contact atom in SiO_2 slab LUMO2, low energy state of contact atom in SiO_2 slab HOMO2, high energy state of the adjacent atom to SiO_2 interfacial atom LUMO3, low energy state of the adjacent atom to SiO_2 interfacial atom HOMO3.

System	LUMO1	HOMO1	LUMO2	HOMO2	LUMO3	HOMO3
C=O/SiO_2-O	3.96727	-0.44773	1.98112	-0.57488	1.98112	-0.57488
OH/SiO_2-O	3.89894	-0.44706	2.14131	-0.47969	2.14131	-0.47969
NH_2/SiO_2-O	4.18587	-0.44713	2.1067	-0.4793	2.1067	-0.4793
SH/SiO_2-O	3.55785	-0.47615	1.15087	-0.89513	1.15087	-0.89513
SiOH/SiO_2-O	5.47309	-0.45591	2.04228	-0.54272	2.04228	-0.54272
C=O/SiO_2-OH	3.93453	-0.47947	8.69812 Pz	-0.58188 pz	9.18312 pz	-0.58188 pz
OH/SiO_2-OH	3.95998	-0.44702	8.70019 pz	-0.51781 pz	9.15219 pz	-0.51781 pz
NH_2/SiO_2-OH	4.14997	-0.47903	8.69899 pz	-0.55001 pz	9.15199 pz	-0.55001 pz
SH/SiO_2-OH	3.60023	-0.44577	8.69974 pz	-0.51726 pz	9.15274 pz	-0.51726 pz
SiOH/SiO_2-OH	5.45002	-0.45698	8.69919 pz	-0.54981 pz	9.15219 pz	-0.54981 pz
C=O/SiO_2-Si	3.96354	-0.44746	3.1261	-0.7019	3.3181	-0.5739
OH/SiO_2-Si	3.95623	-0.44677	3.31839	-0.57461	3.31839	-0.57461
NH_2/SiO_2-Si	4.21116	-0.47884	3.28671	-0.57429	3.31871	-0.54229
SH/SiO_2-Si	3.56781	-0.44619	3.31814	-0.60586	3.31814	-0.57486
SiOH/SiO_2-Si	5.43416	-0.45484	3.28641	-0.57459	3.35041	-0.54259

Table S2. ΔLUMO1 is the difference of the high energy state between the molecule contact atom and SiO_2 contact atom, which is defined as ΔLUMO1=LUMO1 − LUMO2. ΔHOMO1 is the difference of the low energy state between the molecule contact atom and SiO_2 contact atom, which is defined as ΔHOMO1=HOMO1 − HOMO2. ΔLUMO2 is the difference of the high energy state between the SiO2 contact atom and the adjacent atom, which is defined as ΔLUMO2=LUMO2 − LUMO3. ΔHOMO2 is the difference of the low energy state between the SiO2 contact atom and the adjacent atom, which is defined as ΔHOMO2=HOMO2 − HOMO3. q is the transferred charge between the interface. $\ln|q|$ is the natural logarithm of the absolute values of q. The sign of the $\ln|q|$ is related its physical meaning and $\pm\ln|q|$ is negatively correlated with q.

System	ΔLUMO1	ΔHOMO1	ΔLUMO2	ΔHOMO2	q	$\pm\ln\|q\|$
C=O/SiO_2-O	1.98615	0.12715	0	0	-0.00162	-6.42533
OH/SiO_2-O	1.75763	0.03263	0	0	-0.05576	-2.8867
NH_2/SiO_2-O	2.07917	0.03217	0	0	-0.01261	-4.37327
SH/SiO_2-O	2.40698	0.41898	0	0	-0.09641	-2.33915
SiOH/SiO_2-O	3.43081	0.08681	0	0	-0.00242	-6.02399
C=O/SiO_2-OH	-4.76359	0.10241	-0.485	0	0.00036	7.92941
OH/SiO_2-OH	-4.74021	0.07079	-0.452	0	0.00232	6.06619
NH_2/SiO_2-OH	-4.54902	0.07098	-0.453	0	0.00204	6.19481
SH/SiO_2-OH	-5.09951	0.07149	-0.453	0	0.00798	4.83082
SiOH/SiO_2-OH	-3.24917	0.09283	-0.453	0	0.00229	6.0792
C=O/SiO_2-Si	0.83744	0.25444	-0.192	-0.128	-0.00234	-6.0576
OH/SiO_2-Si	0.63784	0.12784	0	0	-0.00098	-6.92796
NH_2/SiO_2-Si	0.92445	0.09545	-0.032	-0.032	-0.00769	-4.86783
SH/SiO_2-Si	0.24967	0.15967	0	-0.031	-0.00045	-7.70626
SiOH/SiO_2-Si	2.14775	0.11975	-0.064	-0.032	-0.00155	-6.4695
